# Hypertension prevalence and risk factors in rural and urban Zambian adults in western province: a cross-sectional study

**DOI:** 10.11604/pamj.2018.30.97.14717

**Published:** 2018-06-05

**Authors:** Kathy Lynn Rush, Fastone Matthew Goma, Jessica Amelia Barker, Rachel Ann Ollivier, Matthew Scott Ferrier, Douglas Singini

**Affiliations:** 1 School of Nursing, University of British Columbia, Okanagan, Kelowna, Canada; 2 Department of Physiological Sciences, The University of Zambia, Lusaka, Zambia; 3 School of Nursing, Dalhousie University, Halifax, Canada; 4 Zambia Ministry of Health, Limulunga District, Zambia

**Keywords:** Hypertension, adult, Zambia, rural, urban, cross-sectional, lifestyle practices, non-communicable diseases, chronic disease

## Abstract

**Introduction:**

Hypertension is a longstanding problem in Zambia, yet little is known about its prevalence and risk factors, particularly in rural and urban settings. Identifying geographical variations in hypertension is important to enhance the health of adult Zambians regardless of where they live. Therefore, the purpose of this study was to compare the prevalence of hypertension and related risk factors between rural (n = 130) and urban (n = 131) communities in Western Province, Zambia.

**Methods:**

This cross-sectional study included urban and rural adult Zambians attending health checks who completed a modified World Health Organization (WHO) survey, and had blood pressure and anthropometric measurements completed. Descriptive statistics were used to summarize demographic and risk factor variables. Chi-square tests of association were used to analyze relationships between categorical variables, t-tests to analyze relationships between continuous variables and logistic regression to examine associations of hypertension with selected risk factors.

**Results:**

The prevalence of hypertension in rural Zambians was double (46.9%) that of urban Zambians (22.9%). Increasing age, not engaging in walking/biking, and alcohol intake within the last 30 days were associated with an increased likelihood of hypertension in rural Zambians while eating vegetables more days during the week was associated with a decreased likelihood of hypertension in rural Zambians. Body Mass Index (BMI) was significantly associated with an increased likelihood of hypertension in urban Zambians.

**Conclusion:**

Modifiable risk factors (such as BMI, dietary intake, and physical activity) are associated with hypertension prevalence in this population, indicating opportunities for screening and other prevention measures.

## Introduction

**Hypertension in Sub-Saharan Africa**: Estimates indicate that hypertension is a widespread problem in sub-Saharan Africa (SSA), although comprehensive data is lacking in many countries. However, the study, prevention, and treatment of hypertension remain low priorities of governing health authorities [1]. Consequently, the true burden of hypertension is unknown [2]. According to United Nations Population Fund data, 125.5 million people are projected to be living with hypertension in SSA by 2025, with the prevalence rate rising to 17.4% from 16.2% in 2008 [3]. Hypertension is the most common risk factor both for cardiovascular disease worldwide, and for stroke, heart failure, and coronary artery disease, specifically in SSA and is the leading contributor to increased premature death and disability [1,4]. There is little information on the prevalence and risk factors for hypertension in Zambia, particularly in rural areas of the country [5]. Few studies have compared rural and urban differences in hypertension prevalence, but identifying such differences is important to enhance the health of adult Zambians regardless of where they live. Therefore, the purpose of this study was to compare the prevalence of hypertension and related risk factors between rural (Limulunga, Miulwe) and urban (Mongu) communities in Western Province, Zambia.

**Rural and urban hypertension prevalence**: Mean systolic and diastolic blood pressure (BP) have been shown to increase with age in both rural and urban populations worldwide [6], with rural populations showing a lower overall prevalence of hypertension [7-9]. In the World Health Survey comparison of hypertension prevalence in rural and urban settings in Africa, South Africa and the Democratic Republic of the Congo showed the greatest contrast with prevalence rates 10 percent higher in urban compared to rural areas [9]. Ethiopia and Tanzania showed the narrowest rural-urban gap with prevalence of hypertension in urban settings only 5 percent higher than in rural settings [9]. In contrast, recent African-based comparison studies of rural and urban populations show that prevalence rates in rural areas are quickly increasing to match that of urban centres, with a higher prevalence of hypertension in rural settings observed in several studies [6,10-12]. In Zambia, studies of hypertension prevalence and risk factors have been sparse until more recently. Oelke et al [13] found a prevalence of 32.8% hypertension in Zambian adults living in Mongu, the urban capital of Western Province. Slightly lower prevalence rates have been reported in rural Zambia. Mulenga et al [5] reported hypertension prevalence of 25.8% in Kaoma and 30.3% in Kasama, two rural districts in Zambia. Similarly, Yan et al [14] reported an age standardized hypertension prevalence rate of 28% in adult Zambians (>25 years) presenting to rural primary health clinics. No Zambian urban-rural comparison studies of hypertension prevalence were found.

**Rural and urban hypertension risk factor comparison**: Rural populations in Africa have been shown to generally be more advanced in age, less overweight or obese, have lower incomes, higher use of smokeless tobacco products, be less educated, more active, and to have a higher prevalence of malnutrition [4,6,11,13,15]. Lower hypertension prevalence in rural African populations have been attributed to lifestyle factors, such as increased daily exercise as a result of their work requirements compared to the slightly more sedentary lifestyles of urban populations [13,16]. Increased adoption of a westernized diet has had a strong influence on both urban and rural populations [11]. Hypertension studies specific to rural Zambia have found similar results. Mulenga et al [5] found hypertension highly associated with age, body mass index (BMI), and smoking in two rural Zambian districts. In Kaoma, adults 45 years and older and with BMI >30 were more likely to be hypertensive. In Kasama, in addition to older age, smoking was found to be significantly associated with hypertension with smokers 21% (AOR=1.21, 95% CI (1.02, 1.45)) more likely to be hypertensive compared to non-smokers. Yan et al (2015) [14] found significant relationships between age and hypertension and BMI and hypertension in rural Zambians. This paper reports on the results of a study of hypertension prevalence and associated risk factors in rural and urban communities in Western Province, Zambia.

## Methods

**Design and setting**: This study used a cross-sectional design to observe differences in BP and related risk factors between Zambian adults in rural (n = 2) and urban (n = 1) communities. The setting for this research was in Mongu, Limulunga, and Miulwe Districts in the Western Province of Zambia. Prior to recruitment and data collection, ethics approval was obtained from the University of British Columbia Okanagan (UBCO) Behavioural Research Ethics Board (H16-03229) and the Biomedical Research Ethics Committee at the University of Zambia (008-01-17). The Zambian Ministry of Health and the National Health Research Authority of Zambia also provided permission to do the research.

**Sampling**: Zambian adults, 18 years of age and older, from one urban and two rural communities, were recruited to participate in the study. Community members were invited to participate in a "Health check" station held in high traffic areas, such as the local markets, bus stations, and grocery stores. Four Health Checks in total were conducted; two in Mongu District and two in Limulunga District. Word-of-mouth recruitment strategies were used, based on their successful use in other recruitment efforts [13]. A sample size of 250, based on approximately 30 participants per variable, was deemed to provide sufficient power to detect significant relationships [17].

**Data collection**: Informed consent was obtained prior to data collection. Participants were informed of their rights to withdraw from the study without penalty; anonymity; and their access to research findings upon study completion. Data collection was completed by faculty instructors and fourth year nursing students from UBCO who were in Zambia for a global health practicum during March-April 2017, and Zambian registered nursing students from Lewanika School of Nursing. Five stations were set up at each Health Check site, with a local Zambian nursing student and a UBCO nursing student. Local health care professionals were onsite, reviewing and referring all participants found to have moderate to severe hypertension and/or a cardiac arrhythmia to the appropriate local health facility for additional follow-up. Although English is prevalent in Western Province, local translators were present at all Health Check stations for lay persons who were more comfortable speaking their local language. Zambian nursing students, proficient in the local language (Silozi), acted as translators and assisted with obtaining consent from participants. A modified version of the standardized World Health Organization (WHO) STEPwise approach to surveillance [18] was used for the collection of quantitative data. The following data were collected: BP, BMI (weight, height), Waist Circumference (WC) and self-reported health behaviours, including alcohol use, smoking, physical activity (PA) and diet. Blood pressure was measured using the 2014 Clinical Practice Guidelines for the Management of Hypertension in the Community by the American Society of Hypertension and the International Society of Hypertension. The guidelines recommend two BP measurements separated by at least one minute, with the average BP calculated from the first and second readings [19]. According to the guidelines hypertension is a systolic BP ≥ 140 mm Hg or a diastolic BP ≥ 90 mmHg, or both. Height, weight and WC were measured using standardized approaches to ensure accuracy and consistency [20]; height and weight measurements were used to calculate BMI (wt(kg)/ht(m)^2^).

**Data analysis**: All quantitative data were entered into an ExcelTM database and imported into the Statistical Package for Social Sciences for Windows (SPSS)TM version 24.0 for analysis. Descriptive statistics were used to summarize demographic and risk factor variables. Chi-square tests of association were used to analyze relationships between categorical variables. T-tests were used to analyze relationships between continuous variables. Logistic regression was used to examine associations of hypertension with the following risk factors: age, sex, BMI, WC, average number of days/week eating fruits and vegetables, alcohol consumption for the past 30 days, walking or use of bike for 10 minutes/day. Odds ratios (unadjusted odds ratios (OR) & adjusted odds ratios (AOR) and their 95% confidence intervals (CI) are reported. The level of significance for all analyses was set at p < 0.05.

## Results

A description of the sample appears in Table 1. There were 261 adults who completed surveys, 131 urban adults and 130 rural adults with 64.8% (n = 169) females and 35.2% (n = 92) males. The study sample consisted of 48.9% (64) male and 51.1% (67) females from urban areas and 21.5% (28) males and 78.5% (102) females from rural areas. Rural participants were significantly older than urban participants, with an average age of 54.13 ± 17.56) (Range: 22-90) and 39.40 ± 14.39 (Range: 18-93), respectively.

**Table 1:** Description of sample

Demographic characteristics		Urban		Rural	
		Frequency	Percent	Frequency	Percent
Age	Age group	Range=18-93; Mean=39.4; SD=14.4		Range=22-90; Mean=54.1; SD=17.6	
	18-29	42	32.5	13	10.3
	30-39	30	23.2	18	14.3
	40-49	26	20.2	16	12.7
	50-59	17	13.3	26	20.6
	60+	14	10.8	53	42.1
Sex	Male	64	48.9	28	21.5
	Female	67	51.1	102	78.5
Marital status	Never married	38	29.0	10	7.7
	Currently married	68	51.9	73	56.2
	Separated or divorced	15	11.5	10	7.7
	Widowed	10	7.6	37	28.5
Education level	No formal schooling	4	3.1	20	15.4
	Less than primary school	11	8.4	40	30.8
	Primary school completed	44	33.6	49	37.7
	Secondary school completed	56	42.7	18	13.8
	College/university completed	14	10.7	3	2.3
	Post-graduate degree	2	1.5	--	--
Work	Government employee	11	8.5	1	0.8
	Non-government employee	17	13.1	1	0.8
	Self-employed	79	60.8	40	30.8
	Unemployed (able to work)	13	10.0	45	34.6
	Other (student, homemaker, retired, unable to work, non-paid)	10	7.6	43	33.0

**Figure 1:**
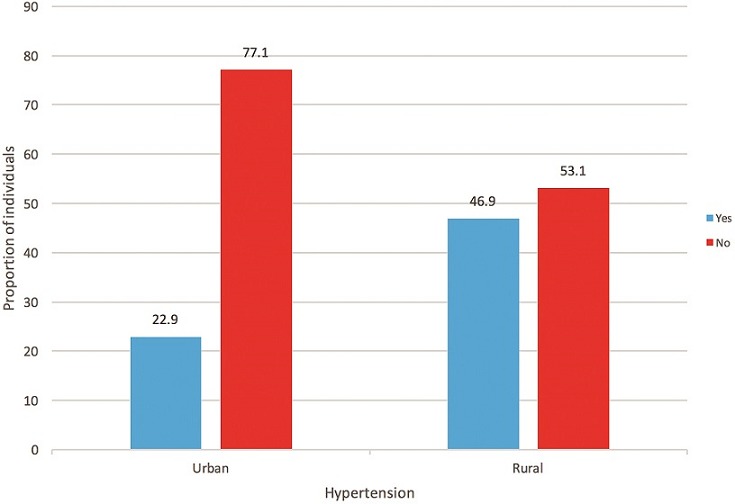
Hypertension prevalence in rural and urban Zambians

**Blood pressure prevalence**: Hypertension prevalence was significantly greater in rural than in urban Zambians (Figure 1). Rural Zambians had a significantly higher average BP of 136.22/79.62 (SD 23.30/12.91) compared to their urban counterparts with an average BP of 127.04/78.36 (SD 15.39/10.36). In contrast, prehypertension was significantly greater in urban Zambians (45.0%, n = 59) than in rural Zambians (23.8%, n=31) (χ^2^=12.971, p = 0.000). Hypertension and pre-hypertension values for urban and rural populations stratified according to age and sex appear in Table 2 and Table 3, respectively. Sixty percent of rural and urban Zambians had had their BP measured at some time during their lifetime by a doctor or health care professional. Significantly more rural Zambians (41.5%, n = 54) had been told they had raised BP compared to urban Zambians (29.0%, n = 38) (χ^2^ = b4.489, p = 0.034), a difference that disappeared when looking at the past 12 months only for being informed about raised BP. Of those who had ever been told they had raised BP (n = 92), 46.7% (n = 43) had normal BP at the time of the Health Check. Of those found to have hypertension (stage 1 or 2) at the health check, 34.1% had never had their BP measured by a doctor or other health provider with no significant difference in previous BP checks between rural and urban Zambians (χ^2^ = 0.011, p = 0.918).

**Table 2:** Blood pressure by age and sex (% of urban population, N=129*)

Blood pressure	18-29 years of age		30-39 years of age		40-49 years of age		50-59 years of age		60 years of age and older	
	Male	Female	Male	Female	Male	Female	Male	Female	Male	Female
**Normal (<130/<80)**	6.2	10.8	3.9	5.4	7.8	3.9	3.1	3.1	<2.5	3.1
**Pre-hypertension (130-139/80-89)**	7.0	3.9	4.7	3.9	<2.5	<2.5	<2.5	<2.5	<2.5	<2.5
**Stage 1 (140-159/90-99)**	<2.5	3.1	<2.5	3.1	<2.5	<2.5	--	<2.5	<2.5	<2.5
**Stage 2 (>159/>99)**	--	--	--	<2.5	<2.5	<2.5	<2.5	<2.5	--	--
**Total**	14.7	17.8	10.8	12.4	13.2	7.0	5.4	7.8	4.7	6.2

* Missing age values for 2 participants

**Table 3:** Blood pressure by age and sex (% of rural population, N=126*)

Blood pressure	18-29 years of age		30-39 years of age		40-49 years of age		50-59 years of age		60 years of age and older	
	Male	Female	Male	Female	Male	Female	Male	Female	Male	Female
**Normal (<130/<80)**	<2.5	6.3	<2.5	9.5	4.8	3.2	--	6.3	3.2	4.0
**Pre-hypertension (130-139/80-89)**	--	<2.5	--	<2.5	--	<2.5	--	4.8	<2.5	<2.5
**Stage 1 (140-159/90-99)**	<2.5	--	<2.5	<2.5	--	<2.5	<2.5	6.3	<2.5	11.9
**Stage 2 (>159/>99)**	--	--	--	<2.5	--	<2.5	--	<2.5	4.8	11.1
**Total**	1.6	8.7	2.4	11.9	4.8	7.9	0.8	19.8	12.7	29.4

* Missing age values for 4 participants

**Demographic factors and blood pressure**: There was no significant difference in systolic BP between men and women in urban or rural groups although urban men had a slightly higher mean systolic BP (128.16 ± 13.47 mmHg) than urban women (125.97 ± 17.06 mmHg) and rural women had a slightly higher mean systolic BP (136.33 ± 22.67 mmHg) than rural men (135.82 ± 22.35 mmHg). Increasing age was associated with an increased likelihood of exhibiting hypertension only in rural Zambians (AOR = 1.076, CI = 1.043-1.110, p = 0.000; OR = 1.059, CI = 1.033-1.086, p = 0.000). Marital status was similar between rural and urban Zambians with more in urban locations (29%) having never been married and more in rural locations who were widowed (28.5%). Rural Zambians showed higher BPs compared to urban Zambians regardless of marital status (e.g, never married, married, widowed/divorced). Education, employment, and earnings differed significantly between rural and urban Zambians. There was a significant difference between rural and urban Zambians in number of years in school with a mean of 10.19 years (SD = 3.55) for urban Zambians and a mean of 6.96 years (SD = 2.94) years for rural Zambians (t = 6.783, p = 0.000). There was a slightly positive, but non-significant relationship between years spent in school and systolic average BP (r = 0.057, p = 0.437). Urban Zambians compared to rural Zambians had completed higher levels of education and had fewer reporting no schooling or only primary school. There was a significant difference between rural and urban Zambians in main work status (χ^2^ = 80.021, p = 0.000). Zambians living in urban areas had higher employment rates (82.3%) than their rural counterparts (32.3%). Thirty-five percent of rural Zambians reported unemployment compared to only 10% of urban Zambians with a significant rural-urban difference both in those unemployed but able to work (χ^2^= 23.016, p=0.000) and those unemployed, but not able to work (χ^2^= 59.005, p = 0.000). There was a significant difference in self-employment between rural (31%) and urban Zambians (60%) (χ^2^= 22.947, p = 0.000) but BP was not significantly different between self-employed urban and rural Zambians. Earnings reflected employment status with rural Zambians reporting significantly lower weekly, monthly, and yearly earnings compared to urban Zambians.

**Hypertension risk factor comparison Anthropometrics**: There was no significant difference in WC between urban (79.86 ± 21.02 cm) and rural (80.54 ± 17.49 cm) Zambians. There was no between group difference in BMI (t = 1.046, p = 0.297) with a mean urban BMI of 24.72 ± 4.49 kg/m^2^ and rural BMI was 25.65 ± 9.15 m/kg^2^. BMI was significantly associated with an increased likelihood of hypertension in urban Zambians (AOR=1.271, CI=1.112-1.452, p = 0.000; OR = 1.247, CI = 1.120-1.388, p = 0.001) but not in rural Zambians.

**Nutrition**: There was a significant difference between rural and urban Zambians in weekly consumption of fruit with 48.5% of rural and 17.1% of urban Zambians reporting they did not eat any fruit during the week (χ^2^=31.820, p = 0.000). Compared to rural Zambians, urban Zambians exceeded intake of fruit for all categories of days they ate fruit (1-2, 3-4, 5-6, >6 days). There was no significant difference in vegetable intake between the two groups, with urban and rural groups comparable both in the numbers of days they ate vegetables and in the number of vegetable servings. However, eating vegetables more days during the week was associated with a decreased likelihood of hypertension in rural Zambians (AOR = 0.765, CI = 0.451-0.945, p = 0.024; OR = 0.770, CI = 0.584-1.016, p = 0.064).

**Physical activity**: Surprisingly, rural Zambians reported spending slightly more time on average sitting or reclining than their urban counterparts: a mean of 5.49 ± 5.06 hours vs. 4.74 ± 4.49 hours, respectively. However, this ¾ of an hour difference was not significant (t = 1.134, p = 0.258). Yet, rural Zambians (72.2%) compared to urban Zambians (52.4%) reported doing significantly more work involving vigorous-intensity activity (e.g., carrying or lifting heavy loads, digging or construction work) for at least 10 minutes continuously (χ^2^= 10.560, p = 0.001). Rural Zambians had a significantly higher mean systolic BP compared to their urban counterparts whether they reported vigorous work (t = 2.926, p = 0.004) or not (t = 2.858, p = 0.005). However, the lack of work-related activity was not associated with hypertension in either group. Rather a lack of engagement in walking or 10 minutes of bike riding was associated with rural Zambians having 4.6 times the odds of having hypertension (AOR = 4.567, CI = 1.227-17.007, p = 0.024; OR = 2.169, CI = 0.794-5.924, p = 0.131).

**Smoking and alcohol consumption intake**: There was no significant difference in use of cigarettes or cigars with approximately equal numbers of urban (90%) and rural (86%) Zambians reporting no use of tobacco products. Alcohol consumption in the last 30 days was not significantly different between rural and urban participants with both groups on average reporting zero drinks in one sitting. However, rural Zambians who consumed alcohol within the past 30 days were 8 times more likely to exhibit hypertension than those who did not consume alcohol (AOR = 8.077, CI = 1.084-60.181, p = 0.041; OR = 2.852, CI =.704-11.559; p=0.142).

## Discussion

A strength of this study is its unique comparison of hypertension prevalence and related risk factors between rural and urban communities in Zambia. A hypertension prevalence in rural Zambians double (46.9%) that of urban Zambians (22.9%), contrasts with numerous studies showing a higher prevalence of hypertension in urban African populations [7-9]. Yet, current findings are comparable to other recent studies indicating the hypertension gap closing between rural and urban areas [6,10-13,15]. Rural hypertension prevalence rates in the current study were substantially higher than those reported in two other studies conducted in rural Zambian communities, in which rates ranged from 25.8% to 30.3% [5,14]. They were also higher than previously reported urban Zambia hypertension prevalence rates, ranging from 32.8% in Mongu, Western Province [13] to 34.8% in Lusaka, Zambia [20]. In contrast was the significantly higher proportion of urban participants (28.9%) with pre-hypertension compared to rural participants. The pre-hypertension prevalence in urban Zambians is higher than the age-standardized prevalence of pre-hypertension in sub-Saharan Africa of 21.0% [2] and above the 24.6% pre-hypertension found in a previous study of adults in Western Province, Zambia [13]. The hypertension-prehypertension trend in rural and urban Zambians paralleled age-related propensities. In the current study, older age was significantly associated with hypertension only in rural Zambians, who were 15 years older than their urban counterparts. A ten-year (spanning 1994-2003) comparison study conducted in rural and urban Cameroon similarly found a higher prevalence of hypertension in rural areas and attributed it to the older average age (47 years) of the rural participants [8]. Rural and urban Zambians had comparable anthropometrics with mean WC and BMI slightly higher in rural Zambians, but only BMI fell in the overweight category [21]. This finding contrasts with the general trend towards obesity observed in urban populations [9,22] and reflects the closing gap between rural and urban Zambians in risk factors for hypertension. Despite similar anthropometrics, it was only in urban Zambians that an increase in BMI was associated with an increased likelihood of hypertension. Goma et al [20] similarly found a greater likelihood of hypertension in urban Zambian adults who had a BMI of > 25 kg/m^2^. In an urban Cameroon study of hypertension and adiposity in which BMI and WC were both found to be predictors of hypertension, for every kg/m*2* increase in BMI the prevalence of hypertension rose by 11%, and for each 1 cm increase in WC there was a 9% increase in hypertension [23]. The higher BMIs and WCs observed in rural Zambians in the current study is consistent with their reportedly greater sedentary activity and low fruit intake. This may reflect high rates of unemployment in rural Zambians, contributing to lower overall earnings and, therefore, less ability to afford more expensive foods such as fruits. Very low intake of fruit and vegetables have been reported in Kenyan and Ugandan populations [24,25]. Of particular concern in the current study was that nearly half (48.5%) of rural Zambians ate no fruit on a weekly basis, a figure nearly three times that of their urban counterparts.

This fruit intake is well below the recommended (WHO) fruit intake of five or more servings on a typical day. It is not uncommon in this rural Western Province for nshima, a staple food made of maize flour and water mixed together and of very low nutritional value, to be consumed daily for all three meals because of its affordability, and in effect substituting for healthier choices such as fruits. Even though vegetable consumption did not differ between urban and rural Zambians, eating vegetables more days during the week was associated with a decreased likelihood of hypertension in rural Zambians. Researchers in another study, similarly found that a significantly higher frequency of fruit/vegetables was associated with lower BPs in rural populations in sub-Saharan Africa [26]. Rural Zambians engaged in significantly more vigorous work than urban Zambians, but also spent on average 45 minutes more per day sitting or reclining than their urban counterparts, although this difference was not significant. Rural Zambians spent a mean of 5.5 hours sitting or reclining, more time than Mulenga and colleagues [5] reported (>3.5 hours) in their hypertension study of rural Zambians, and consistent with the > 4-fold increase in hypertension that was only observed among rural Zambians who did not engage in walking or 10 minutes of bike riding. These findings are comparable to those from a rural Ugandan study in which 29.8% of their rural population had low PA levels, despite the presence of vigorous work with many of the study participants being farmers [25]. However, current findings contrast with the lower prevalence of hypertension associated with work-related PA found in Africans from three rural communities [26]. The high percentage of self-employed rural Zambians, compared to urban Zambians, in the current study may have created demands so great that time for physical activity/exercise was limited. There were no differences between urban and rural populations in their level of smoking or alcohol consumption. When comparing these findings to other studies, alcohol consumption has shown an increase over the past decade in both rural and urban areas in SSA [8]. Despite the lack of rural-urban difference in alcohol consumption, an unexpected finding was that rural Zambians who consumed alcohol within the past 30 days were 8 times more likely to exhibit hypertension than those who did not consume alcohol.

This contrasts with another study of rural Zambians that found no significant association of alcohol intake and hypertension in rural Zambians [5]. A weakness of the study was its focus on Western Province, Zambia limiting generalizability to other parts of Zambia or SSA. In addition, the sample size was not large. Further the sample was not randomly generated with the potential of selection bias; perhaps those who were ill tending to stop by the health checks. Although trained fourth year nursing students who were supervised by UBCO nursing faculty assisted with data collection, it is possible that some variation may have occurred with this approach. Self-reported data may have influenced reliability; however, direct measures of BP and anthropometric measures were obtained. Age-adjusted hypertension rates to account for the different age distributions between rural and urban Zambians were not possible due to unknown proportions for hypertensive patients according to age but might have allowed for more equitable comparisons. Future research should replicate these comparative findings on a larger scale by expanding into other rural and urban communities in Western Province. Additionally, longitudinal studies that evaluate trends in hypertension/prehypertension prevalence and risk factors are needed as is research examining the impact of hypertension screening on risk factor identification and short and long term management among Zambian adults.

## Conclusion

Overall, prevalence of hypertension was greater in rural Zambians and they had a higher burden of risk factors for hypertension-age, anthropometrics, nutrition, and physical activity-compared to their urban counterparts. This coupled with the concerning higher incidence of prehypertension in younger, urban Zambians (mean age of 39 years) warrants attention to early screening and detection. The critical need for routine and standardized hypertension and prehypertension screening is reinforced by current findings that 34% of Zambians (urban and rural) confirmed to have hypertension (stage 1 or 2) at the Health Check had never had their BP measured by a doctor or other health provider. Evidence shows that in Western Province, as well as many other countries in sub-Saharan Africa, screening for hypertension is not happening routinely [27]. The lack of basic screening equipment, such as functional sphygmomanometers and stethoscopes, points to the need for policy-level change that prioritizes increased availability of diagnostic equipment for the detection of hypertension/pre-hypertension. On average, policy implementation in Zambia takes two years and has been identified as a challenge in terms of combating non-communicable diseases, such as hypertension, in the country [28]. The importance of follow-up treatment and education are imperative to promote self-management and address non-modifiable risk factors such as physical activity, nutrition, weight, and alcohol intake all found to be predictors of hypertension in the current study. At a national level, it would be useful for policy-makers to incorporate an upstream approach to NCD prevention that promotes healthy lifestyles and decision-making, such as proper, simple to understand nutritional labelling [18,27].

### What is known about this topic

- Hypertension is a widespread problem in sub-Saharan Africa (SSA) although the true burden of hypertension is unknown;
- Data on hypertension prevalence and risk factors for hypertension in Zambia, and in rural areas are limited;
- There is a dearth of studies comparing rural and urban differences in hypertension prevalence and risk factors in Zambia.

### What this study adds

- The prevalence of hypertension in rural Zambians was double (46.9%) that of urban Zambians (22.9%);
- In rural Zambians, increased age, lack of leisure physical activity, and alcohol intake within the last 30 days were associated with an increased likelihood of hypertension while eating vegetables more days during the week was associated with a decreased likelihood of hypertension;
- In urban Zambians, increased BMI was significantly associated with an increased likelihood of hypertension.
